# Congenital Heart Disease Increases Mortality in Neonates With Necrotizing Enterocolitis

**DOI:** 10.3389/fped.2018.00312

**Published:** 2018-10-23

**Authors:** Ulf Kessler, Eva-Maria Hau, Marcin Kordasz, Stephanie Haefeli, Catherine Tsai, Peter Klimek, Dietmar Cholewa, Mathias Nelle, Mladen Pavlovic, Steffen Berger

**Affiliations:** ^1^Department of Pediatric Surgery, Inselspital, Bern University Hospital, University of Bern, Bern, Switzerland; ^2^Center of Visceral Surgery, Bern, Switzerland; ^3^Department of Pediatrics, Inselspital, Bern University Hospital, University of Bern, Bern, Switzerland; ^4^Department of Pediatric Surgery, Cantonal Hospital Aarau, Aarau, Switzerland

**Keywords:** necrotizing enterocolitis, NEC, congenital heart disease, CHD, outcome, mortality

## Abstract

**Background:** Studies on the influence of congenital heart disease (CHD) on neonates with necrotizing enterocolitis (NEC) have produced varied results. We therefore examined the influence of CHD on NEC outcomes.

**Methods:** We carried out a retrospective single-center study including infants with confirmed NEC, treated between 2004 and 2017. We excluded patients with isolated patent ductus arteriosus or pulmonary hypertension (*n* = 45) and compared outcomes of patients with hemodynamically relevant CHD (*n* = 38) and those without CHD (*n* = 91).

**Results:** Patients with CHD were more mature than those without CHD [gestational age, median, 95% confidence interval (CI95), 37.1, 34.5–37.2w, vs. 32.6, 31.9–33.3w; *P* < 0.01]. The presence of CHD did not influence the frequencies of severe disease (overall 21% Bell stage III), nor surgical interventions (overall 30%), the occurrence of intestinal complications (overall 13%), nor the duration of hospitalization (overall 38 days in survivors). The overall mortality as well as NEC-related mortality was increased with the presence of CHD, being 50% (19 out of 38) and 13% (5 out of 38), respectively, when compared to patients without CHD, being 8% (7 out of 91) and 3% (3 out of 91). The presence of CHD and of advanced NEC stage III were independent predictors of NEC-associated fatalities with multivariable odds ratios (CI95) of 7.0, 1.3–39.5 for CHD, and of 3.4, 1.6–7.5 for stage III disease.

**Conclusions:** While some outcome parameters in neonates with NEC remained unaffected by the presence of CHD, the mortality risk for patients with CHD was seven times higher than without CHD.

## Introduction

The presence of congenital heart disease (CHD) has an unclear impact on overall morbidity and mortality in neonates suffering from necrotizing enterocolitis (NEC), with prior studies having conflicting results. A study carried out by Pickard et al. showed the somewhat surprising result (1) that patients with NEC who also suffered from CHD had a significant survival advantage. While this was a relatively large study, there was a notable heterogeneity among the patient groups and its transferability is an area of concern. In addition, these results could not be supported by other publications claiming a worse outcome of NEC in the presence of CHD (2, 3).

We suspect that in the study carried out by Pickard et al. a disproportionate number of NEC severities between the study groups (NEC without CHD vs. NEC with CHD) might have influenced the study outcome, thus resulting in better outcomes for patients with both NEC and CHD. The latter group comprised of more patients with suspected NEC (29 vs. 21%) and less patients with advanced NEC (22 vs. 44%) (1).

Meanwhile, other studies argued that infants with both NEC and CHD had worse outcomes than patients who had only one of the two diseases (2, 3). In the study by Ostlie et al. the number of infants with NEC and CHD was very small (*n* = 6), Fisher et al. on the other hand, only included patients with a birth weight below 1,500 g. In addition, it is not apparent from these studies if patients died due to NEC or due to other causes. Finally, except in the study from Pickard et al. there are currently no other studies addressing intestinal complications such as ileus or short gut syndrome.

Our aim was to gain more insight into the influence of CHD on patients suffering from NEC. We therefore compared outcomes between NEC patients with and without CHD, only including confirmed cases of NEC and assessing intestinal complications.

## Materials and methods

We performed a retrospective review of the institutional database of the Bern University Hospital and identified infants who suffered from NEC between December 2004 and May 2017. The local ethics committee approved our study project. We first excluded patients with isolated patent ductus arteriosus (PDA), pulmonary hypertension, spontaneous intestinal perforation, and suspected NEC (Bell stage I) (4). We previously published the outcome data from a cohort of infants who had NEC and PDA (5). We performed Bell staging based on patient chart review, including radiographies by two out of four independent senior physicians (UK, MN, PK, SB) (4), and resolved disagreement by discussion in all cases. Based on the most recent echocardiography report before the diagnosis of NEC, we subdivided CHDs into different categories.

We defined the following target variables: overall mortality, mortality due to NEC, disease severity (Bell stage), surgical intervention for NEC, duration of hospital stay, occurrence of a relevant intestinal complication like the recurrence of NEC, stricture or stenosis, intestinal failure, defined as prolonged parenteral nutrition (>40 days), short gut syndrome, or—if relevant—others. In addition, we extracted the following data: gestational age (GA, weeks, w), birth weight (BW, grams, g), gender, Apgar scores, age at disease onset (d), and routine laboratory parameters at disease onset and at maximum deviation from the normal value during the acute phase of NEC.

Death was assumed to be the consequence of NEC when the treating physicians primarily attributed death to acute worsening of NEC, and not to cardiac disease.

We used SPSS version 21 (IBM, SPSS, Chicago, IL, USA) for the statistical analysis. We tested data for normality and equal distribution via analysis of skewness and kurtosis and performed the Kolmogorov-Smirnov test. We also made comparisons between groups using student's *t*-test, non-parametric tests or analysis of variance (ANOVA) (as required, respectively) for continuous variables as well as Chi-square or Fisher's exact test (as required, respectively) for categorical variables.

To assess the influence of CHD and disease severity, we carried out univariable and multivariable logistic regression analysis for each outcome parameter. We adjusted multivariable regression for BW, gestational age, and postnatal age at onset of NEC. Data is given as median and 95% confidence interval of the mean (CI95) for continuous variables, and as percentage and frequency for categorical variables unless otherwise specified. We used two-sided tests throughout. *P* < 0.05 was considered significant.

## Results

**Figure 1:**
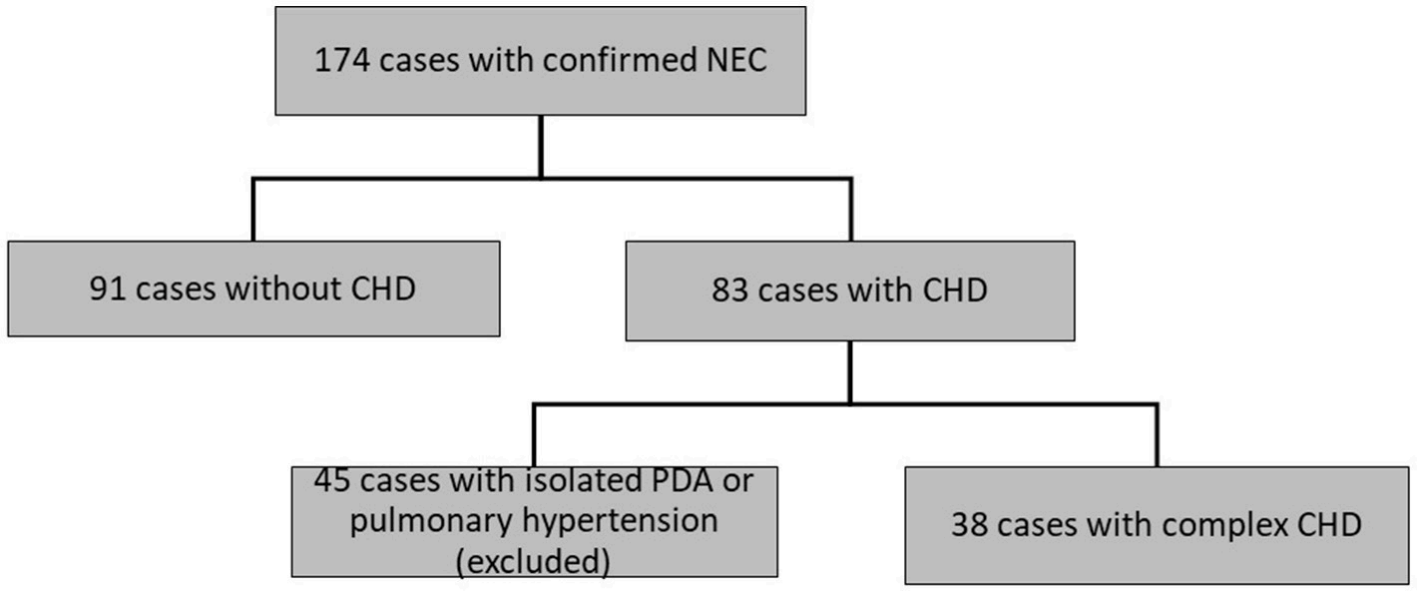
Flow chart of selection of patient with NEC with or without CHD. NEC, necrotizing enterocolitis. CHD, congenital heart disease. PDA, patent ductus arteriosus.

Of 174 patients included in this study with confirmed NEC, 45 infants had isolated PDA or pulmonary hypertension and therefore had to be excluded. Ninety-one infants with NEC had no CHD and 38 infants with NEC had a relevant CHD (see Figure 1, Table 1). Gestational age and BW were significantly higher in the patients with CHD (median, CI95:37.1, 34.5–37.2w; 2,483, 2086–2634 g) than in those without CHD (32.6, 31.9–33.3w; 1,700, 1633–1938 g; *P* < 0.01, respectively) (see Table 2).

**Table 1:** Types and frequencies of CHDs in the group of 38 infants with complex CHD and NEC.

**CHD–type**	***n***	**Additional defects**
ASD	3	
Aortic stenosis	2	
AVSD	6	1 pulmonary atresia
Cardiomyopathy with systolic dysfunction	1	
Coarctation of aorta	3	1 AVSD
Double outlet right ventricle	1	1 VSD, ASD, AS, AR
Hypoplastic aortic arch	1	1 tricuspid atresia
Hypoplastic left heart syndrome	1	
Interrupted aortic arch	1	1 ASD, VSD
PDA	4	2 ASD, 1 PHT, 1 VSD
Tetralogy of Fallot	2	1 AVSD
Total anomalous pulmonary venous return	2	1 TGA, AVSD; 1 ASD
Transposition of the great arteries	3	1 PDA, ASD; 1 AVSD
Tricuspid atresia	2	1 VSD; 1 pulmonary atresia, ASD
Truncus arteriosus	3	1 insufficiency of truncal valve, 1 ASD
VSD	3	1 ASD, 1 PDA, 1 PFO

*AR, aortic regurgitation; AS, aortic stenosis; ASD, atrial septal defect; AVSD, atrioventricular septal defect; PDA, patent ductus arteriosus; PFO, persistent foramen ovale; PHT, pulmonary hypertension; TGA, transposition of the great arteries; VSD, ventricular septal defect*.

**Table 2:** Baseline cohort data.

**Variable**	**NEC without CHD (*N* = 91)**	**NEC with CHD (*N* = 38)**
Male gender, *n (%)*	46 (51%)	22 (58%)
Gestational age (w), median, CI95	32.6 (31.9–33.3)	37.1 (34.5–37.2)*
Birth weight (g), median, CI95	1700 (1633–1938)	2483 (2086–2634)*
Apgar 1', median, CI95	6.0, 5.4–6.3	6.0, 4.8–6.6
Apgar 5', median, CI95	8.0, 7.4–8.1	8.0, 6.6–8.2
Apgar 10', median, CI95	9.0, 8.3–8.9	8.0, 7.4–8.7
Age at diagnosis of NEC (d), median, CI95	6.0, 8.2–12.0	7.5, 10.1–23.6

* *P < 0.01*.

While all laboratory parameters significantly changed during the acute phase of the disease in both patient groups, we nevertheless found some relevant differences between them: hemoglobin concentrations were lower at baseline and during disease in subjects with CHD as compared to those without CHD (*P* < 0.05, respectively) (see Table 3). In addition, patients with CHD had higher baseline levels of CRP as well as higher excess levels of CRP (see Table 3).

**Table 3:** Routine laboratory parameters at disease onset and their maximum abnormality during disease.

**Variable**	**NEC without CHD:Value at disease presentation**	**NEC without CHD:Excess value during disease**	**NEC with CHD:Value at disease presentation**	**NEC with CHD:Excess value during disease**	***P* for differences between groups at disease presentation**	***P* for differences of excess values between groups**
Hemoglobin (G/l), median, CI95	152, 148–161	123, 121–132	136, 126–146	110, 101–116	*P* < 0.05	*P* < 0.01
Platelet count (G/l), median, CI95	251, 242–301	186, 172–225	189, 177–273	107, 106–197	NS	NS
WBC count (G/l), median, CI95	9.0, 10.0–14.3	14.9, 17.0–21.9	9.0, 8.4–13.0	17.9, 16.2–22.9	NS	*P* < 0.05
CRP (mg/dl), median, CI95	5, 9–22	33, 44–73	27, 27–67	102, 80–148	NS	*P* < 0.05
Lactate concentration (mg/dl), median, CI95	2.1, 2.1–3.0	2.3, 2.6–3.6	2.3, 2.4–5.0	3.4, 3.3–7.5	NS	*P* < 0.05
Base excess (mmol/l), median, CI95	−1.3, −0.8 to −2.5	−2.7, −2.3 to−4.1	−3.8, −1.9 to−5.1	5.5, −4.5 to 10.0	NS	*P* < 0.01

*Laboratory changes within groups between onset and maximum abnormality was significant at a P < 0.05 for all laboratory parameters in both groups, respectively. NS, not significant; WBC count, white blood cell count; CRP, C-reactive protein*.

There was a trend toward increased disease severity (Bell stage 211 III) in NEC with CHD (29%) as compared to NEC without 212 CHD (18%; overall 21%). The frequency of surgical intervention 213 (overall 30%), the duration of hospitalization (38 d in survivors) 214 and occurrence of intestinal complications (overall 13%) were comparable in the two groups (see Table 4).

**Table 4:** Risk of outcomes in subjects with CHD and without CHD.

**Variable**	**NEC without CHD (*N* = 91)**	**NEC with CHD (*N* = 42)**	**Univariable OR, CI95; *P***	**Multivariable OR, CI95; *P***
Severe disease, Bell stage III, *n* (%)	16 (18%)	11 (29%)		
Surgery for NEC, *n* (%)	26 (29%)	13 (34%)		
Overall mortality, *n* (%)	7 (8%)	19 (50%)*		
NEC- related mortality, *n* (%)	3 (3%)	5 (13%)*	7.4, 1.6–33.5; *P* < 0.05	7.0, 1.3–39.6; *P* < 0.05
Intestinal complications in survivors, *n* (%)	11 (13%)	2 (11%)		
Duration of hospital stay in survivors, median, CI95	38d, 39–60 d	50d, 41–69 d	–	–

CI95, 95% confidence interval; ICU, intensive care unit; OR, odds ration

* *P < 0.05*.

Overall mortality was lower in patients who had no CHD (7 out of 91, 8%) as compared to the subjects that had CHD (19 out of 42, 50%; *P* < 0.01). NEC-associated mortality was also lower in the patients without CHD (3 out of 91, 3%) as compared to the patients with CHD (5 out of 42, 13%; *P* < 0.05), resulting in an elevated odds ratio for NEC-attributable fatality if CHD was present (univariable OR 7.4, 95% confidence interval 1.6–33.5; *P* < 0.01; multivariable OR 7.0, 95% CI 1.3–39.6; *P* < 0.05, respectively). The second independent risk factor for NEC-attributable fatality was high severity of disease (Bell stage III) (univariable OR 3.2, 95% CI 1.6–6.4; *P* < 0.01; multivariable OR 3.4, 95% CI 1.6–7.5; *P* < 0.01). Comparing the individual subgroups of CHD patients in terms of outcomes, no differences could be identified (data not shown).

## Discussion

The main result of the present investigation is that patients with confirmed NEC had higher rates of overall and of NEC-related mortality if they additionally suffered from CHD. Our results of worse outcome in NEC patients with CHD compared to patients without CHD are not contradictory to the above-cited results of Pickard et al. Firstly, while Pickard et al. included infants with suspected NEC (at an unbalanced ratio of 29% in infants with CHD vs. 21% in infants without CHD), we only assessed outcomes in patients with confirmed NEC (1). Secondly, while Pickard et al. had a lower number of grade III NEC infants in CHD patients (22%) than in patients without CHD (44%), we could show a comparable rate of severe diseases (Bell stage III) in both groups (1).

The poor outcome for NEC patients with concomitant CHD has already been described in a number of studies, but these also included patients with Bell stage I and lacked a comparison with the data provided by NEC patients without CHD (6, 7). Furthermore, these studies only included pre-term infants (8) or patients who were treated surgically (9).

A significantly higher mortality in NEC patients with CHD has been shown in a prospective study by Fisher et al. with a stringent NEC definition and a very specific comparison. However, this study only concerned VLBW infants with a gestational age of 30 weeks or younger (3). Increased mortality in children with CHD (and also with late onset of NEC), has been shown by Short et al. However, this study only included full-term infants (10).

A further study suggested that the outcome of NEC in pre-term and full-term infants might be similar, using a cohort of 277 patients with various gestational age. This relatively large population however only included 26 full-term patients and only six of them suffered from CHD. Consequently, no specific comparison between patients with NEC and CHD and NEC without CHD was possible and no significant difference in mortality could be shown (2). Hence, the present study is, to the best of our knowledge, the first investigation comparing patients with established NEC (Bell stage ≥ II) without CHD and NEC patients with CHD, including both pre-term and full-term patients.

We were able to show that some laboratory parameters differed significantly in children with CHD and those without. Children with a concomitant CHD had a lower white blood cell (WBC) count and higher C-reactive protein (CRP) values as well as lower base excess. The exact significance of this difference remains unclear. While animal studies have shown that polymorphonuclear neutrophils play an important role in the pathogenesis of NEC (11), it is still not clear if local (intestinal) pathology can be demonstrated by blood analyzes. Previous attempts to find a correlation between higher CRP or WBC count and disease severity in NEC have been hampered (12), but there is a scale to assess the severity of NEC, which uses WBC count and base excess as predictors, thus treating neutropenia and low base excess as additional risk factors (13). It has also been shown that lower WBC count correlates with poor outcome (14). CRP, on the other hand, is used as a marker of imminent complications (15). Overall, the difference in WBC count and CRP as well as base excess could reflect the disease severity in CHD patients. However, further research is needed to clarify this.

The hemoglobin values were also lower in children with CHD. It has been shown that anemia is a risk factor for the development of NEC (16). Children with lower hematocrit also tend to have a higher risk for transfusion-associated NEC (17). Hence, the lower concentration could contribute to disease severity. That said, children with CHD were also older and could have lower hemoglobin concentrations due to their gestational age.

A third result of our study is that although mortality is increased in patients with CHD, surviving patients do not have more intestinal complications than patients without CHD do. This is an important finding, in line with the findings of Pickard et al. because, as we could recently show in a meta-analysis, gastrointestinal sequelae are a frequent problem, which should not be underestimated when assessing disease outcome (18).

### Strengths

This is, to the best of our knowledge, the second study to compare intestinal outcomes in NEC patients with and without CHD. Including pre-term as well as full-term patients and a BW over 1,500 g, this study also draws on the biggest patient cohort. Furthermore, the fact that we only included patients with confirmed NEC is certainly a great strength of this study. In this way, we could reduce the potential bias of deviation toward better outcomes in patient groups with suspected NEC. Finally, we performed a thorough review of patient charts, including many variables including ones that allowed us to assess NEC-related mortality and intestinal complications.

### Shortcomings and limitations

Our study certainly has some limitations. The retrospective study design engenders several potential sources for bias. In addition, the long inclusion period of more than 12 years bears a multitude of possible confounders influencing the results, for example, the changing intensive care protocols, different feeding habits or new surgical approaches. However, we are convinced that the duration of inclusion has a relatively small influence on the assessed outcome parameters because the year of birth was not correlated with mortality rate in our cohort. A comparatively stable mortality rate of 360 NEC patients in the period from 1986 to 1999, as reported by Luig and Lui (19), supports our result of a stable mortality rate over time (19).

It can be suggested that some subgroups of CHD even cause worse NEC outcomes as compared to others, but there is still no proof for this assumption (3). Since we only examined a relatively small number of CHD patients, we were unable to confirm or refute this hypothesis.

Unfortunately, it was not possible for us to determine if patients with CHD were treated more aggressively than those without CHD. However, since all included patients received clinical and radiological workup as well as withdrawal of enteral feeds and intravenous antibiotic treatment at disease onset, it is very likely that initial patient management has little impact on the outcome.

Finally, there is also some risk of bias in our definition of mortality. In the absence of objective criteria, we assumed that patients died due to NEC whenever the treating physicians claimed so. However, it is likely that the health condition of patients with co-morbidities might have deteriorated due to cardiac causes or others and not due to enterocolitis itself. Unfortunately, it was not possible to evaluate this retrospectively. Nevertheless, the diagnostic criterion stated by the physicians is widely used, also in studies particularly evaluating the mortality (20). Finally, while most of the existing studies are rather non-specific, our study is one of the few that also defines the cause of death (2, 3).

## Conclusion

We conclude that patients with established NEC and CHD are more mature and have a seven-fold increased mortality risk in comparison to NEC patients without CHD. Our results also suggest that CHD-associated NEC could be seen as a separate entity, with a different pathogenesis than classical NEC. Furthermore, we believe that special care must be taken and aggressive treatment must be considered in the management of NEC patients with a concomitant CHD because of its much poorer outcome.

## Ethics statement

The protocol was approved by the Kantonale Ethikkommission Bern, Switzerland.

## Author contributions

UK, E-MH, PK, DC, and SB contributed to the conception and design of the work. UK, E-MH, MK, SH an MN contributed to the acquisition, and analysis of data. All authors contributed to the interpretation of data for the work. All authors drafted the work or revised it. All authors approved the final version and agreed to the publication. All authors agree to be accountable for all aspects of the work.

### Conflict of interest statement

The authors declare that the research was conducted in the absence of any commercial or financial relationships that could be construed as a potential conflict of interest.
